# An integrated signature of extracellular matrix proteins and a diastolic function imaging parameter predicts post-MI long-term outcomes

**DOI:** 10.3389/fcvm.2023.1123682

**Published:** 2023-04-12

**Authors:** Hiromi W.L. Koh, Anna P. Pilbrow, Sock Hwee Tan, Qing Zhao, Peter I. Benke, Bo Burla, Federico Torta, John W. Pickering, Richard Troughton, Christopher Pemberton, Wern-Miin Soo, Lieng Hsi Ling, Robert N. Doughty, Hyungwon Choi, Markus R. Wenk, A. Mark Richards, Mark Y. Chan

**Affiliations:** ^1^ Department of Medicine, Yong Loo Lin School of Medicine, National University of Singapore, Singapore, Singapore; ^2^ Institute of Molecular and Cell Biology (IMCB), Agency for Science, Technology and Research (A*STAR), Singapore, Singapore; ^3^ Department of Medicine, Christchurch Heart Institute, University of Otago, Christchurch, New Zealand; ^4^ National University Heart Centre, National University Health System, Singapore, Singapore; ^5^ Singapore Lipidomics Incubator (SLING), Life Sciences Institute, National University of Singapore, Singapore, Singapore; ^6^ Precision Medicine Translational Research Programme and Department of Biochemistry, Yong Loo Lin School of Medicine, National University of Singapore, Singapore, Singapore; ^7^ Heart Health Research Group, University of Auckland, Auckland, New Zealand

**Keywords:** integrative analysis, multi-omics, echocardiogaphy, major adverse cardiac events (MACE), heart failure hospitalization

## Abstract

**Background:**

Patients suffering from acute myocardial infarction (AMI) are at risk of secondary outcomes including major adverse cardiovascular events (MACE) and heart failure (HF). Comprehensive molecular phenotyping and cardiac imaging during the post-discharge time window may provide cues for risk stratification for the outcomes.

**Materials and methods:**

In a prospective AMI cohort in New Zealand (*N* = 464), we measured plasma proteins and lipids 30 days after hospital discharge and inferred a unified partial correlation network with echocardiographic variables and established clinical biomarkers (creatinine, c-reactive protein, cardiac troponin I and natriuretic peptides). Using a network-based data integration approach (iOmicsPASS+), we identified predictive signatures of long-term secondary outcomes based on plasma protein, lipid, imaging markers and clinical biomarkers and assessed the prognostic potential in an independent cohort from Singapore (*N* = 190).

**Results:**

The post-discharge levels of plasma proteins and lipids showed strong correlations within each molecular type, reflecting concerted homeostatic regulation after primary MI events. However, the two molecular types were largely independent with distinct correlation structures with established prognostic imaging parameters and clinical biomarkers. To deal with massively correlated predictive features, we used iOmicsPASS + to identify subnetwork signatures of 211 and 189 data features (nodes) predictive of MACE and HF events, respectively (160 overlapping). The predictive features were primarily imaging parameters, including left ventricular and atrial parameters, tissue Doppler parameters, and proteins involved in extracellular matrix (ECM) organization, cell differentiation, chemotaxis, and inflammation. The network signatures contained plasma protein pairs with area-under-the-curve (AUC) values up to 0.74 for HF prediction in the validation cohort, but the pair of NT-proBNP and fibulin-3 (*EFEMP1*) was the best predictor (AUC = 0.80). This suggests that there were a handful of plasma proteins with mechanistic and functional roles in predisposing patients to the secondary outcomes, although they may be weaker prognostic markers than natriuretic peptides individually. Among those, the diastolic function parameter (E/e' - an indicator of left ventricular filling pressure) and two ECM proteins, *EFEMP1* and follistatin-like 3 (*FSTL3*) showed comparable performance to NT-proBNP and outperformed left ventricular measures as benchmark prognostic factors for post-MI HF.

**Conclusion:**

Post-discharge levels of E/e', *EFEMP1* and *FSTL3* are promising complementary markers of secondary adverse outcomes in AMI patients.

## Introduction

Circulating proteins and lipids are clinically useful biomarkers for the diagnosis and prognosis of cardiovascular diseases (CVD) (1–3). Historically, prognostic biomarker studies have favored an analytic framework focusing on one or a few prioritized candidates within a risk stratification framework to predict specific clinical events (4–7). Recently, omics-scale technologies such as mass spectrometry, affinity-based proteomics, and nuclear magnetic resonance spectroscopy have enabled simultaneous measurement of hundreds to thousands of analytes including proteins, metabolites, and lipids in cardiovascular research (8–11).

Despite the arrival of high-throughput platforms, the new discoveries have barely challenged the use of imaging parameters to aid detection of abnormalities in the heart and biomarkers of heart failure (HF) and that of traditional biomarkers such as NT-proBNP and cardiac troponin-I/T to evaluate cardiac injury, and these two modalities firmly remain as the gold standard clinical practice for long-term prognostication. In addition, although the number of assessable analytes increased with omics scale assays, few studies have carefully investigated the relationship between newly measured circulating molecules and the conventional cardiac markers and imaging parameters.

A possible explanation for this phenomenon is that the emerging plasma proteomics and metabolomics data sets are often analyzed from the perspective of the predictive capacity alone in biomarker studies, and that the natriuretic peptides and troponins often outperformed most challengers in terms of diagnostic or prognostic indicators as they represent the most tissue specific evidence of myocardial damage. As a result, most data analyses tend to overlook the massive correlation structure underlying the milieu of measured molecules and neglect the biologically interpretable, meaningful co-variations in these rich data sets.

We have previously reported an analysis of plasma protein candidates predicting post-MI heart failure events (HF) in two independent post-MI cohorts in a similar context, with unique complementary information from a single cell transcriptomics data set generated for mouse heart (12). Expanding on this work, we put together a systems-level data analysis approach to integrate plasma lipids, acylcarnitines, proteins and multi-parametric cardiac imaging data, with the express purpose of building an interpretable prognostic signature of long-term post-MI major adverse cardiovascular events (MACE) and HF. Our key approach is the use of network topology information as adjudicating evidence for biomarker prioritization, in combination with the conventional univariate prognostic potential measured by the area under the curve (AUC) of the receiver operating characteristic (ROC).

Given the highly correlated nature of multi-dimensional data across the different modalities, we improved our previously published data integration approach iOmicsPASS (13) and present the new toolbox as iOmicsPASS + . Using the new tool, we first identify an undirected graph, or a partial correlation network of all data features *via* estimation of a sparse precision matrix (14) and then discern subnetwork signatures predictive of clinical outcomes using a network-based scoring approach. Using the clinical outcome data collected prospectively and molecular data profiled at 30 days after hospital discharge, we searched for the prognostic network signatures of HF as well as other MACE. To isolate pure predictive signals within the intra- and inter-modality correlation structures and ensure the compatibility of information between the training and test/validation cohorts, we performed the network-level analysis without incorporating other conventional demographic risk factors such as age and gender.

## Materials and methods

### The coronary disease cohort study (CDCS)

The CDCS cohort consisted of 2,140 patients recruited from two tertiary hospitals (Christchurch Hospital and Auckland City Hospital) in New Zealand (NZ) for an acute coronary syndrome (ACS) event from 2002 to 2009 (ACTRN 12605000431628). Participants with angiographically-documented coronary artery disease were invited to return to the hospital 30 days after discharge for baseline biometric, echocardiographic and blood-based measurements. Patients were excluded from the study if their life expectancy was estimated to be less than 3 years. More information on the study can be found in Prickett et al. (15). Details of the nested case-control cohort (*N* = 741) and supervised analysis on the subcohort (*N* = 464) are in the **Supplementary Information**.

### The improving outcomes in myocardial infarction through reversal of cardiac remodelling (IMMACULATE) cohort

The IMMACULATE cohort consisted of 859 patients who were admitted for MI at 3 tertiary hospitals in Singapore (National University Heart Centre, Tan Tock Seng Hospital and National Heart Centre) from 2011 to 2014. The patients were followed up for a median of 3.9 years (interquartile range IQR: 2.0–4.8 years) from their hospital discharge date, and biometric, echocardiographic, and blood-based measurements were collected at the same post-discharge time point (30 days) as the CDCS. Detailed information on the nested case-control subset 190 patients used as a validation cohort in this report can be found in the **Supplementary Information**.

### Proteomics, lipidomics and clinical biomarker measurements

Protein abundance was measured using Slow Off-rate Modified Aptamer (SOMAmer)–based capture array, called SOMAscan® (SomaLogic, Inc, Boulder, CO, United States) (16) and reported as relative fluorescent units (RFU). Targeted lipidomics experiments were performed using an Agilent 6495A triple quadrupole (QQQ) mass spectrometer coupled to an Agilent 1,290 Infinity-II UHPLC system, with automated data processing and quality control. Acylcarnitines were also measured as part of this panel. One lipid standard was used for each lipid class and the ratio of the peak areas of endogenous lipids to their respective lipid standards was reported as the lipid measurements (without conversion to molar concentrations). Please refer to Section 2 of the **Supplementary Information** for the details of data acquisition and quality control.

Established cardiac markers, including natriuretic peptides (ANP, BNP, NT-proANP, NT-proBNP), high sensitivity troponin-I (hsTNI) and creatinine, were measured using clinical-grade assays. In CDCS, the concentrations of natriuretic peptides were measured in pmol/l except for NT-proANP (in nmol/L). In IMMACULATE, the concentrations of ANP, BNP and NT-proBNP were measured in pg/ml and NT-proANP was not measured. To allow for comparison between the two studies, we unified the units of measurements to pg/ml for ANP, BNP and NT-proBNP and ng/ml for NT-proANP. For ANP and NT-proANP, we converted 1 pmol/L to 3.081 pg/ml and 1 nmol/l to 12.7 ng/ml, respectively. For BNP and NT-proBNP, we converted the units using 1 pmol/l to 3.47 pg/ml and 1 pmol/l to 8.475 pg/ml, respectively. Due to the higher accuracy and sensitivity of the clinical-grade assays, we removed four corresponding proteins measured in SOMAscan® (**Supplementary Information**).

### Transthoracic echocardiography

Standard M-mode measurements of left ventricle (LV) dimensions, wall thickness and left atrial (LA) dimensions were made according to the recommendations of the American Society of Echocardiography (ASE) (17). LV volumes and the derived left ventricular ejection fraction (LVEF) were measured by the Simpson modified biplane method (18). Pulsed-wave Doppler velocities of trans-mitral early diastolic (E) and atrial (A) filling were obtained from the apical 4-chamber view with a 5 mm sample volume placed between the tips of the mitral leaflets (19), and systolic (S), diastolic (D), and atrial reversal (AR) pulmonary vein velocities were acquired from the apical 4-chamber view with a 5 mm sample volume placed 1 cm into the right upper pulmonary vein. Early (e') and late (a') diastolic, and systolic (s') pulsed-wave tissue Doppler velocities were obtained at both septal and lateral mitral annular corners, and averaged (20). The ratio of trans-mitral E and annular e’ velocities (E/e') was derived as a measure of LV filling pressure. Only variables with at least 70% of the measurements quantified were used and missing values in those variables were imputed together with missing entries from the six cardiac markers using multiple imputation by chained equations (MICE) (21). All missing data were assumed to be missing at random (MAR) and the estimates from the multiple models are pooled using Rubin's rule (22).

### Ascertainment of clinical events

The composite outcome of interest was 5-point MACE (or MACE including HF), defined as: MI, stroke, unstable angina, HF and/or a CV-related death. All patients were followed from the time of their primary hospital discharge to a future major adverse cardiac event, death or end of study, whichever was earlier. For both CDCS and IMMACULATE, we defined three phenotypic outcomes: (1) patients who remained event-free (Event-free), (2) patients who had any one of the five adverse cardiac outcomes (MACE), with further stratification by (3) patients with non-fatal and fatal HF events (HF).

### Statistical analysis

For continuous variables, two-sample *t*-tests were used to compare the difference in means across groups, whereas for categorical variables, Chi-squared tests were applied to test for associations with groups. Fisher-exact test was used where cell-frequencies were less than five. Clinical variables with missing or unknown self-reported entries were removed before performing the statistical test for association. All *p*-values were adjusted for multiple-testing correction using the Benjamini-Hochberg's (BH) method to control the overall false discovery rates (FDR). The AUC of the ROC was computed for each marker to evaluate their predictive performance and presented with a 95% confidence interval. Cox proportional hazard (PH) models were used to assess the prognostic value of individual markers from hospital discharge to MACE or HF, death or end of follow-up, whichever is earlier. Hazard ratio (HR), *p*-value, Harrell's C-index and its 95% confidence interval were reported for each marker. Log-rank test was employed to compare the Kaplan-Meier (KM) survival curves when stratified by risk groups.

### Network-level predictive analysis using iOmicsPASS+

To estimate the Gaussian graphical model (GGM) underlying the observed data (with a corresponding partial correlation network), we utilized all markers across 741 patients from CDCS and computed a covariance matrix using all pairwise complete observations as an input to the graphical LASSO algorithm (14), using a module built in iOmicsPASS + . Originally developed as iOmicsPASS for the integration of DNA, mRNA and protein-level multi-omics data over biological networks (13), iOmicsPASS + has been extended to directly infer an undirected graph representing conditional (in)dependence relationships among all continuously scaled data features from the multiple data types, through estimation of GGM (Supplementary Figure S1) (14). Following the network inference, iOmicsPASS is employed to carry out dimension reduction of the information from multiple data sets by using edge-level co-expression scores, taking the direction of the correlation into account, to identify a sparse set of subnetwork signatures that separates the phenotypic groups *via* a supervised approach (see Supplementary Material).

To prevent potential confounding, we removed the patients with remodelled heart, medical history of HF, stroke and previous MI events that occurred within the last five years, leaving 464 CDCS patients for the predictive analysis. We sought to identify subnetwork signatures that best (1) differentiate patients destined to incur secondary MACE from those spared such events, and (2) patients suffering secondary HF from those not, using 10-fold cross-validation (CV) for parameter optimization. Within the iOmicsPASS framework, network signatures are characterized by group-specific centroids of edge-wise co-expression scores. For every *i*-th edge with a non-zero partial correlation estimate, *co-expression scores* were calculated for individual patients. The sign of the partial correlation is reflected into the co-expression score calculation, where the scores for two positively correlated nodes and two negatively correlated nodes are calculated as products and ratios of the normalized data in each subject (sums and differences in logarithmic scale), respectively.

Once subject-level co-expression scores are calculated, test-statistics contrasting group differences, denoted by d_ik_* (23) (*k *= 1 for outcome and *k *= 0 for no outcome), are computed for the edges. For simplicity, we refer to these network edge-wise test-statistics as *d-scores* hereafter, the magnitude of which indicates the combined discriminative power of a pair of predictors in separating the phenotypes. The test-statistics were penalized iteratively to select the optimal sparse network classifiers that minimize the overall misclassification error in the CV. The final *d-scores* are visualized in the networks, incorporating the sign and magnitude, reflected by the color and thickness of edges in the network respectively (13).

iOmicsPASS + also offers a direct prediction functionality on external data sets with the same input data types, allowing for missing data features in the new data set. The prediction module takes all available variables of the selected classifiers from a data set, standardizes the variables within the new data, computes the co-expression scores of predictive edges and derives the composite discriminant scores and classification probabilities for individual patients. These discriminant scores or class probabilities can then be used to stratify patients in the external data, or within the training data itself if necessary.

Further details of the method and the software can be found in the **Supplementary Information**. All analyses were carried out in R version 4.2.0 (24) using in-house R-scripts. Network visualization is done using the Cytoscape software (25).

Throughout this paper, we used the CDCS cohort as the training dataset to build a network and estimate predictive network signatures for both MACE and incident HF. Then using these signatures, we computed the class probabilities on both CDCS and another independent cohort, IMMACULATE, as a validation dataset.

### Secondary analysis of individual markers in the network signatures

As these network signatures were derived without any consideration for time to event, we further assessed if the predicted class probabilities could predict the risk of future MACE and incident HF in the presence of other factors influencing long-term outcomes. We achieved this by fitting three types of Cox PH models: without any clinical adjustments (Model A), adjusted for age, gender and BMI (Model B) and further adjusted for ST-elevation status, history of hypertension and medications prescribed at discharge (Model C). We first identified the medications that were directly associated with either MACE or HF, at both discharge and admission, for adjustment in the model. In CDCS, we adjusted for beta-blockers, ACE inhibitors, aspirin, clopidogrel, calcium channel antagonists, long-acting nitrates, diuretics, statins and warfarin use at hospital discharge. In IMMACULATE, we adjusted for beta-blockers, ACE inhibitors and antagonist receptor blockers (ARB). To determine the usefulness of adding clinical variables to the model, we also calculated the continuous net reclassification index (cNRI) and integrated discrimination index (IDI) of the 2-year event prediction of Model B and Model C, relative to Model A.

## Results

### Characteristics of the CDCS cohort

**Figure 1:**
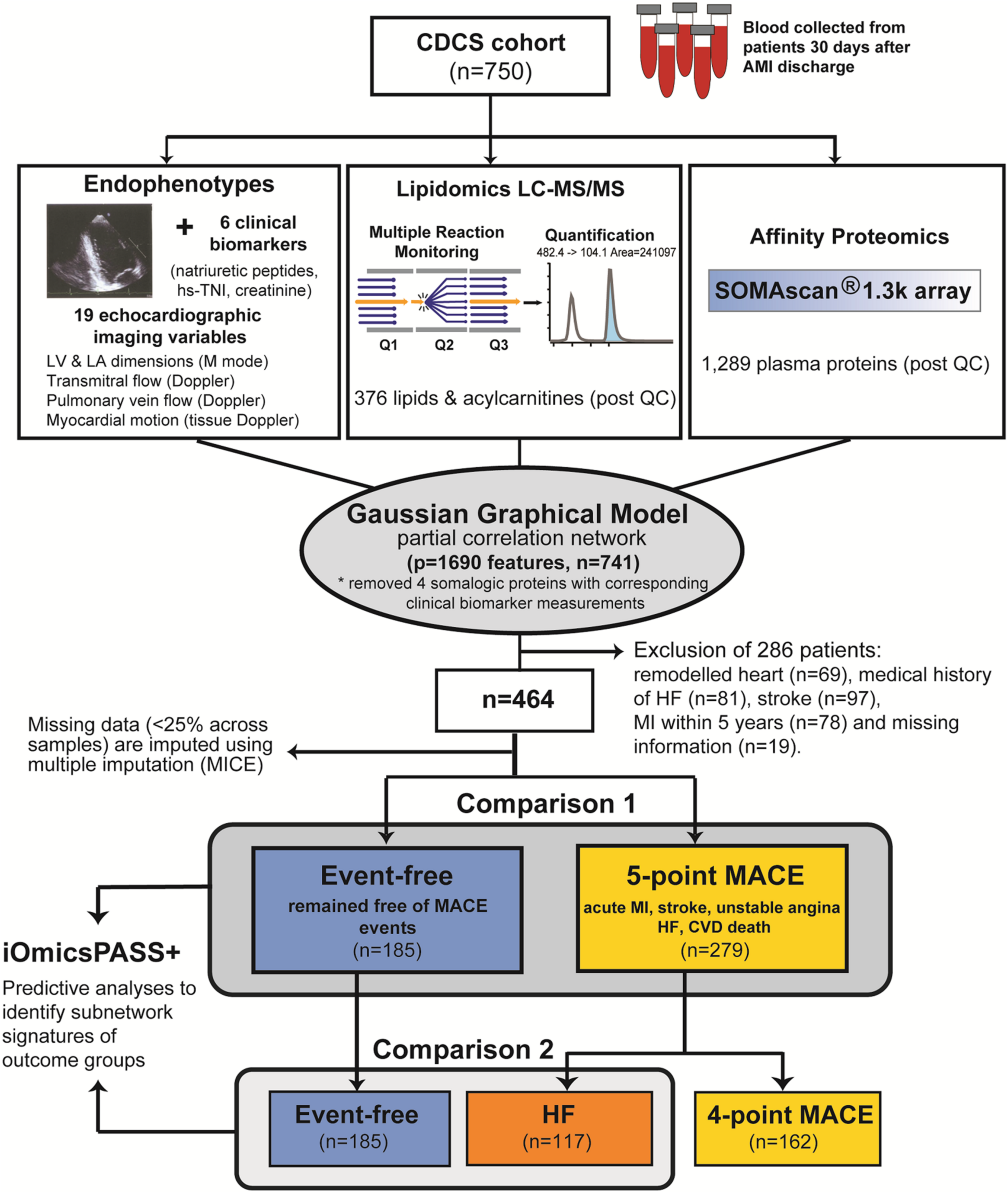
Analysis workflow applied to the CDCS cohort. The background network reflecting the conditional dependence structure among the four data types was inferred using graphical LASSO. From the network, subnetwork signatures of secondary MACE and HF were obtained using the training dataset (CDCS) using 10-fold cross validation for choosing the optimal regularization parameter.

Figure 1 illustrates the analysis workflow for the CDCS cohort. Using the implementation in iOmicsPASS+, we integrated the data for proteins, lipids, clinical biomarkers and echocardiographic measurements from 741 subjects, and derived a network of conditionally dependent data features using the graphical LASSO (26). Given the diversity of data types, we call individual variables “data features” hereafter. For this network, we calculated partial correlation for each edge of the network as a measure of association strength between the two corresponding data features, accounting for the effects of all other variables (see **Supplemental Information**). In the second step, we subsequently identified predictive subnetwork signatures of secondary adverse outcomes, namely MACE and HF. This supervised analysis was conducted using 464 patients meeting the criteria for outcome data (**Supplemental Information**), where 185 patients remained event-free, and 279 patients had a secondary MACE, including 117 patients hospitalized for HF during follow-up. In what follows, we first describe the predictive potential of individual data features and the overall network structure and present the network-driven analysis results afterwards.

Table 1 provides the overall characteristics of the 464 patients. The mean age was 69 years (SD = 10.7 years), with more males (69.2%) than females (30.8%). The majority were of European descent from New Zealand (56.7%) and other countries (27.8%), while the rest (7.1%) were Asians, Africans, Maoris, Fijians and the Pacific Islanders. Most patients either quit smoking (54.5%) or had never smoked (38.8%). Upon admission for a primary AMI episode, 29.7% were diagnosed with STEMI and 70.3% NSTEMI. The median follow-up time from the date of hospital discharge was 4.88 years, and the time from discharge to a MACE ranged from one day to 8.8 years (median 0.74 years). Comparisons between patients remaining event-free and those incurring secondary MACE revealed significant differences in age, diagnosis of ST-segment elevation and hypertension, as well as clinical biomarkers such as serum creatinine and plasma natriuretic peptides. MACE cases were older (mean age = 70.6 years, SD = 11.1) and more frequently hypertensive (56.6%) than patients who remained event-free (36.8%). The index events were also more likely NSTEMI (74.6%) than STEMI (63.8%). Event-free patients had lower levels of serum creatinine and plasma concentrations of natriuretic peptides (ANP, BNP, NT-proANP, NT-proBNP) than those with MACE on follow-up.

**Table 1:** Clinical characteristics of post-AMI patients in CDCS study.

	CDCS study (New Zealand)			
	All	Event-free	MACE	*P*-value
	(*n* = 464)	(*n* = 185)	(*n* = 279)	
**Follow-up time, median (yrs)**	4.88	4.84	4.96	
**Time to MACE, median (yrs)**		–	0.74	
**Age (yrs), mean (SD)**	69 (10.7)	66.7 (9.6)	70.6 (11.1)	<0.001
**BMI (kg/m^2^), mean (SD)**	27.1 (4.9)	27 (3.9)	27.2 (5.5)	0.603
Gender, *n* (%)				
*Males*	321 (69.2)	126 (68.1)	195 (69.9)	0.760
*Females*	143 (30.8)	59 (31.9)	84 (30.1)	
Ethnicity, *n* (%)				
*NZ European*	263 (56.7)	102 (55.1)	161 (57.7)	0.485
*Other Europeans*	129 (27.8)	42 (22.7)	87 (31.2)	
*Others*	33 (7.1)	12 (6.5)	21 (7.5)	
*Unknown*	39 (8.4)	29 (15.7)	10 (3.6)	
Smoking status, *n* (%)				
*Current Smoker*	31 (6.7)	13 (7)	18 (6.5)	0.318
*Ex-Smoker*	253 (54.5)	93 (50.3)	160 (57.3)	
*Never Smoked*	180 (38.8)	79 (42.7)	101 (36.2)	
Alcohol consumption, *n* (%)				
*Current drinker*	294 (63.4)	127 (68.6)	167 (59.9)	0.070
*Ex-drinker*	52 (11.2)	14 (7.6)	38 (13.6)	
*non-drinker*	118 (25.4)	44 (23.8)	74 (26.5)	
ST-elevation status, *n* (%)				
*ST-elevated MI, STEMI*	138 (29.7)	67 (36.2)	71 (25.4)	0.017
*Non ST-elevated MI, NSTEMI*	326 (70.3)	118 (63.8)	208 (74.6)	
**Family history of CAD, *n* (%)**	185 (39.9)	80 (43.2)	105 (37.6)	0.345
**Diabetes Mellitus, *n* (%)**	82 (17.7)	27 (14.6)	55 (19.7)	0.197
**Hypertension, *n* (%)**	226 (48.7)	68 (36.8)	158 (56.6)	<0.001
**Hyperlipidemia, *n* (%)**	209 (45)	82 (44.3)	127 (45.5)	0.687
Medication use at discharge, *n* (%)				
*Beta-blockers*	412 (88.8)	174 (94.1)	238 (85.3)	0.006
*ACE-inhibitors*	280 (60.3)	118 (63.8)	162 (58.1)	0.256
*Aspirin*	456 (98.3)	183 (98.9)	273 (97.8)	0.486
*Angiotensin II type 1 receptor*	19 (4.1)	4 (2.2)	15 (5.4)	0.099
*Clopidogrel*	302 (65.1)	140 (75.7)	162 (58.1)	<0.001
*Calcium channel antagonists*	99 (21.3)	23 (12.4)	76 (27.2)	<0.001
*Long-acting nitrates*	87 (18.8)	22 (11.9)	65 (23.3)	0.003
*Diuretics*	112 (24.2)	27 (14.6)	85 (30.6)	<0.001
*Warfarin*	36 (7.8)	8 (4.3)	28 (10)	0.038
*Statins*	426 (91.8)	175 (94.6)	251 (90)	0.108
Clinical- biomarkers^†^, mean (SD)				
*Creatinine, mg/dl*	99.4 (59.7)	90.3 (18.7)	106 (75.2)	<0.001
*High-sensitive Troponin I (hsTNI), ng/l*	57.8 (506.6)	18.3 (51.5)	84.6 (654.0)	<0.001
*Atrial natriuretic peptide (ANP), pg/ml*	146 (98.8)	126 (76.5)	159 (109.4)	<0.001
*N-terminal pro ANP, ng/ml*	18.2 (13)	14.7 (8.8)	20.6 (14.7)	<0.001
*Brain natriuretic peptide (BNP), pg/ml*	107 (116.1)	76.5 (81.4)	127 (130.6)	<0.001
*N-terminal pro BNP, pg/ml*	1,330 (1539.8)	925 (908.6)	1,600 (1795.6)	<0.001

^†^ Markers were measured one month from hospital discharge post MI.

CDCS, coronary artery disease cohort study; CAD, coronary artery disease; NZ, New Zealand; BMI, body mass index; MI, myocardial infarction; HF, heart failure; LVEF, left ventricular ejection fraction; STEMI, ST-elevated myocardial infarction; NSTEMI, non ST-elevated myocardial infarction; LDL, low-density lipoprotein; HDL, high-density lipoprotein; hsTNI, high-sensitive Troponin I; NT-proANP, N-terminal pro-hormone atrial natriuretic peptide; NT-proBNP, N-terminal pro-hormone brain natriuretic peptide.

Supplementary Table S1 shows the comparison of the echocardiographic measurements between event-free patients and MACE patients, as well as between event-free patients and HF patients. As expected, more echocardiographic variables differed between HF patients and event-free patients than between all MACE patients and event-free patients. Both adverse outcome groups (MACE and the HF subgroup) had greater baseline LA and LV dimensions but lower LVEF than event-free patients. Tissue Doppler parameters of myocardial motion differed between MACE/HF and event-free cases with e' and E/e’ differing most sharply between the subgroup with HF and event-free patients.

### Single markers of MACE and HF

We next compared the post-discharge levels of individual proteins, lipids, echocardiographic parameters and clinical biomarkers in the patients with MACE and HF to event-free patients. A total of 184 data features were significantly different in mean abundance between MACE patients and event-free patients (FDR < 0.05), where 66.3% were higher in MACE patients. Comparing the HF patients with the event-free ones, 368 markers were significantly different (FDR < 0.05). Details of this analysis are reported in Supplementary Table S2.

The differential features of MACE included 166 proteins, seven lipids, five echocardiographic measurements, and all six clinical biomarkers. Although all natriuretic peptides, hsTNI and creatinine levels were higher in MACE patients, BNP and NT-proBNP attained the AUC of 0.65 (95% CI: 0.60–0.70 for BNP and 0.59–0.70 for NT-proBNP), indicating modest discrimination for MACE. Echocardiographic measurements such as LA area, LV mass, indexed left ventricle internal dimension in diastole (LVIDDi) and systole (LVIDSi), as well as indices of diastolic dysfunction (E/e'), were all higher in MACE than in event-free patients, yet the AUC values were modest at best. When considering lipids, only a handful of lipids showed statistically significant differences. Three species (phosphatidylethanolamine PE 34:1 and PE 34:2, sphingosine-1-phosphate S1P d18:0) were higher, whereas four species (phosphatidylcholine PC 38:4 and 40:8, LysoPC 20:4, sphingomyelin SM 43:1) were lower in the MACE patients. Lastly, the top three markers were all plasma proteins, including macrophage-capping protein (*CAPG*) with AUC of 0.68 (95% CI: 0.63–0.73), aspartate aminotransferase (*GOT1*) with AUC of 0.65 (95% CI: 0.60–0.70) and follistatin-related protein 3 (*FSTL3*) with AUC of 0.64 (95% CI: 0.59–0.69).

The 368 differential features of HF included 298 proteins, 31 lipids, nine echocardiographic variables, and all six clinical biomarkers. Similar to the differential features of MACE, all six clinical biomarkers were higher in HF than in event-free patients as expected: NT-proBNP demonstrated the highest AUC of 0.79 (95% CI: 0.74–0.84), followed by BNP with AUC of 0.78 (95% CI: 0.73–0.83). In echocardiographic parameters, all five measurements significantly altered in MACE also differed in the HF subgroup. In addition, interventricular septum (IVS), LA width, LVESVi were also significantly higher, while the early diastolic mitral annulus velocity e’ was significantly lower. Similar to the MACE comparison, the differences in the lipid levels were not pronounced in the HF group, including glycerophospholipids (8 PEs, 3 PC, 4 phosphatidylinositol PI), four lysophospholipids (3 LysoPC, 1 LysoPE), eight sphingolipids (SM 38:1, 38:2, 43:1, 44:1, 44:2), S1P d18:0, ceramide d19:1/24:0 and ganglioside GM3 d18:1/16:0, cholesteryl ester CE 20:4 and two glycerolipids (diacylglycerol DG 38:6 and triacylglycerol TG 58:10). Among those, LPC 20:4, PE 34:2 and PE 35:2 had the highest AUCs at a modest value of 0.64 (95% CI: 0.57–0.71).

Of the 298 significant proteins, 61.7% were higher in HF patients than in event-free patients. *CAPG* had the highest AUC of 0.77 (95% CI: 0.72–0.83), followed by *FSTL3* with AUC of 0.75 (95% CI: 0.70–0.81) and Cystatin-C (*CST3*) with AUC of 0.74 (95% CI: 0.68–0.79). The top performing proteins such as *CAPG*, *CST3*, *FSTL3*, and EGF-containing fibulin-like extracellular matrix protein 1 (*EFEMP1*), also known as fibulin3, were equally predictive of HF as the natriuretic peptides, with overlapping 95% CIs.

### Partial correlation network connecting the four modalities

Supplementary Figure S1 illustrates the network inference workflow of iOmicsPASS + . First, each data type was standardized by mean centering and pareto variance scaling. Then, outlier observations were filtered out before being concatenated into a single data matrix using all 741 samples. The network estimation module produced a network of 27,334 edges in this data set (1.9% of all possible edges) based on the regularized parameter selected at the lowest eBIC value, connecting 1,690 data features. To assign measures of strength to the selected edges, the regularized precision estimates were converted to partial correlations, denoted by *r*, which ranged from −0.43 to 0.66 in this data. We remark that all correlations reported in this work refer to regularized partial correlations, and for this reason, the magnitude of partial correlations may seem small.

Not surprisingly, the intra-modality partial correlations (within the same data type) were much stronger than the inter-modality partial correlations (between data types): only 5.4% of the edges were between proteins and lipids, and as few as 373 edges were between proteins and echocardiographic parameters and clinical biomarkers. This result clearly shows that the proteomic variation is largely independent of the lipidomic variation and imaging parameter differences, and both types of molecular data relate differently to the risk of future adverse outcomes.

**Figure 2:**
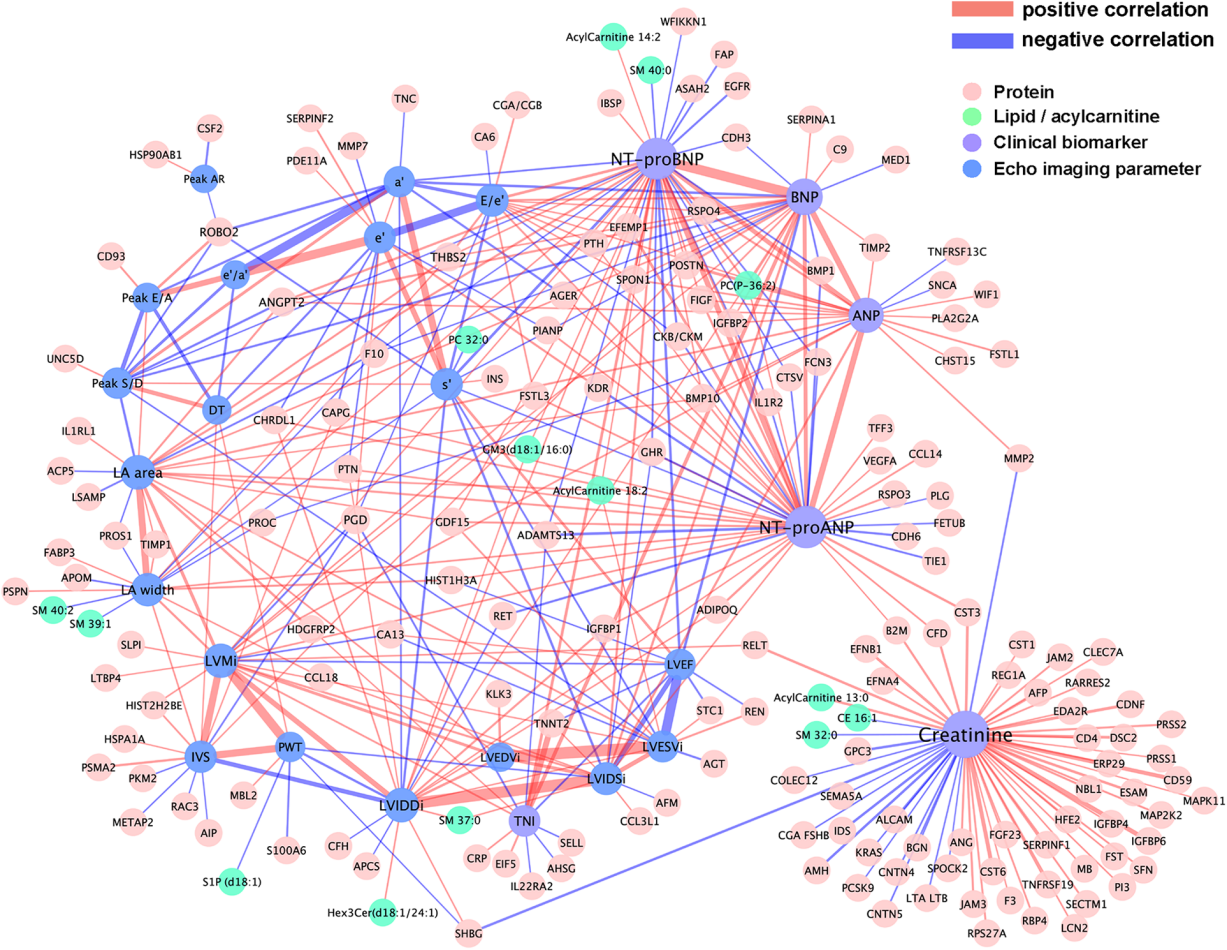
Visualization of the partial correlation network connecting plasma proteins and lipids with echocardiographic imaging variables and cardiac biomarkers. Plasma proteins and lipids were drawn as nodes in light red and cyan, and echocardiographic variables and cardiac biomarkers in blue and light purple, respectively. Network edges were colored according to the signs of partial correlations, i.e. positive in red and negative in light purple.

The core segment of the network connecting echocardiographic measurements and clinical biomarkers to proteins, lipids, and acylcarnitines is shown in Figure 2. In this partial correlation network, the natriuretic peptides and hsTNI (TNI in purple color) were positively correlated with each other and BNP, NT-proBNP and hsTNI were negatively correlated with LVEF. Among the echocardiographic measurements, LVEDVi and LVESVi had the highest partial correlation (*r *= 0.475), followed by LVIDSi with LVIDDi (*r *= 0.459) and the negative correlation between LVEF and LVESVi (*r *= −0.375). Overall, only ten echocardiographic parameters (LA area, LA width, LVEF, LVMi, LVIDDi, LVIDSi, Peak S/D, a', s’ and E/e') were connected with clinical biomarkers in this subnetwork. Among those, the highest correlations were recorded between NT-proBNP and E/e’ (*r *= 0.058), between NT-proBNP and s' (*r *= −0.057), between BNP and LVEF (*r *= −0.053) and between BNP and a' (*r *= −0.050). This finding suggests that myocardial motion parameters captured by tissue Doppler imaging are particularly correlated with neurohormonal activation from the primary MI episodes.

Meanwhile, stronger correlations were observed among proteins, lipids, and acylcarnitines with clinical biomarkers than with echocardiographic parameters, suggesting that morphological and functional features provide orthogonal information to molecular markers during the early recovery post-discharge. The highest correlation among the connected proteins was between hsTNI and cardiac troponin T (*TNNT2*) (*r *= 0.206), followed by the correlation of creatinine with insulin-like growth factor binding protein 2 (*IGFBP2*) (*r *= 0.142), glycoprotein *CD59* (*r *= 0.118), and cystatin M (*CST6*) (*r *= 0.115). Nine proteins were connected with at least three natriuretic peptides in the network, including positive correlation with angiopoietin-2 (*ANGPT2*), periostin (*POSTN*), R-spondin 4 (*RSPO4*), *IGFBP2*, thrombospondin 2 (*THBS2*), Spondin-1 (*SPON1*), vascular endothelial growth factor D (*VEGFD)* (also known as *FIGF*), bone morphogenetic protein 10 (*BMP10*), and negative correlations with bone morphogenetic protein 1 (*BMP1*) and coagulation factor×(*F10*). See Supplementary Table S3 for the table of regularized partial correlation estimates among the data features with the clinical biomarkers from the four modalities.

### Predictive subnetwork signature of MACE

**Figure 3:**
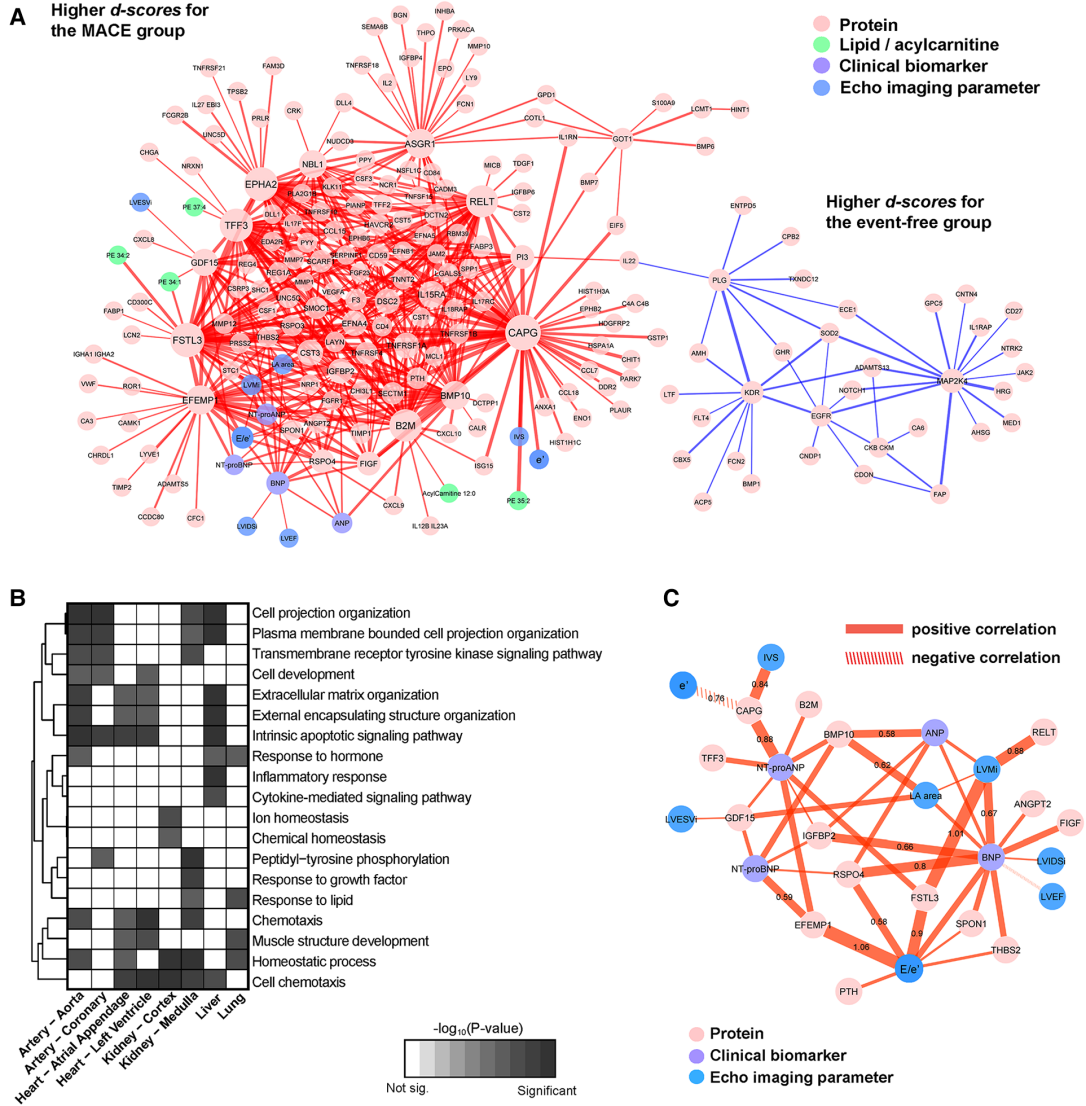
Subnetwork signature of MACE. (**A**) Network visualization with plasma proteins, lipids and acylcarnitines, echocardiographic imaging variables, and clinical biomarkers in different colors. Edges were colored according to the signs of the *d-scores* computed by iOmicsPASS + and the thickness reflects the magnitude of the scores representing the discriminatory power. (**B**) Enrichment of biological functions in the constituent nodes in panel (**A**), accounting for tissue-specific expression of genes at the mRNA level (median-normalized TPM > 5 in each tissue). (**C**) Visualization of the subset of the network signature illustrating the plasma proteins connected to echocardiographic imaging variables and clinical biomarkers.

Using this estimated network as the background, we next applied the supervised analysis module of iOmicsPASS + for dimension reduction and to obtain subnetwork signatures of MACE (13). Table 2 reports the number of proteins, lipids, echocardiographic variables, and clinical biomarkers in the predictive signatures. The MACE signature included 524 edges connecting 211 nodes (mean cross-validated error 38.5%). Figure 3 visualizes the MACE signature, where the edges were colored by the sign of the *d-scores* for the MACE group (red if higher in MACE, blue otherwise). This subnetwork signature contains 194 proteins, five lipids, eight echo imaging parameters and all four natriuretic peptides. Although only five lipids were part of this network, four of them happen to be phosphatidylethanolamines (PE 34:1, 34:2, 35:2, 37:4).

**Table 2:** Summary of the predictive subnetwork signatures of lipids, proteins and clinical markers in two comparisons, separating event-free post-MI patients from those with a future major adverse cardiac outcome (MACE) and from HF patients.

	MACE from event-free patients	HF from event-free patients	Overlaps	Common features	Features unique to MACE	Features unique to HF
Number of predictive network edges	524	566	414			
Total number of features	211	189	160			
Mean cross-validation error	38.5%	26.7%				
**Proteins**	**194**	**164**	**143**			
Over-expressed in at least one tissue	123	109	91			
*Heart-enriched* ^‡^	4	4	4	BMP10, CSRP3, FABP3, TNNT2	–	–
*Artery-enriched* ^†^	7	5	4	EFEMP1, IGFBP2, TIMP1, THBS2	BGN, CNTN4, INHBA	POSTN
*Skeletal muscle-enriched*	5	5	5	CA3, CSF3, CSRP3, FABP3, SOD2	–	–
*Kidney-enriched* ^#^	5	6	4	GDF15, MMP7, SPP1, TDGF1	TNFSF15	IL1RL1, REN
*Liver-enriched*	13	9	8	ASGR1, C4A/C4B, CCL15, CPB2, HRG, IL27, PLG*	AHSG, EPO, FABP1, FCN2, THPO	CLEC4M
*Lung-enriched*	12	15	11	CCL18, CD4, CD300C, CHIT1, CSF3, CST5, CXCL8, CXCL9, CXCL10, FIGF, RSPO4	ACP5	AGER, CD55, CD93, IL1RL1
**Lipids**	**5**	**8**	**5**			
*Phosphatidylethanolamine*	4	5	4	PE 34:1, PE 34:2, PE 35:2, PE 37:4	–	PE(O-36:4)
*Phosphatidylcholine*	0	1	0	–	–	PC(*P*-30:0)
*Acylcarnitines*	1	2	1	AcylCarnitine C12:0	–	AcylCarnitine C14:0
**Echo imaging variables**	**8**	**12**	**8**	IVS, LVEF, LVMi, LA area, LVESVi, LVIDSi, E/e’, e'	–	LA width, LVEDVi, LVIDDi, A'
**Clinical- biomarkers**	**4**	**5**	**4**	ANP, NT-proANP, BNP, NT-proBNP	–	hsTNI

*Plasminogen (PLG) was detected twice; one as the complete plasminogen and the other as angiostatin, a proteolytic fragment.

^#^Tissues enriched in the kidney include cortex and medulla.

^†^ Tissues enriched in the artery include aorta and coronary artery.

^‡^ Tissues enriched in the heart include atrial appendage and left ventricle.

Bold values represent the main features part of the network.

The subnetwork consists of two segments. The majority (96.4%) were highly correlated proteins, lipids, imaging parameters and clinical biomarkers with higher *d-scores* (in red) for the MACE group. The other part was a protein-only network with lower centroids (in blue). Here, the main driver of separation between the two groups were the edges connecting one protein to another (90.6%). Twenty-two edges connected natriuretic peptides to plasma proteins, 13 connected echocardiographic variables to proteins, and six linked lipids to proteins. The edges with the highest *d-scores* for MACE were the joint performance of *CAPG* with several proteins including trefoil factor 3 (*TFF3)*, *FSTL3* and ephrin type-A receptor 2 (*EPHA2)*. The top five highest *d-scores* for the event-free group were between mitogen-activated protein kinase kinase 4 (*MAP2K4*) with prolyl endopeptidase (*FAP*), superoxide dismutase in mitochondria (*SOD2*), histidine-rich glycoprotein (*HRG*) and between plasmin (*PLG*) and vascular endothelial growth factor receptor 2 (*KDR*). Six major hub proteins, *CAPG*, *FSTL3*, *EPHA2*, *TFF3*, tumor necrosis factor receptor superfamily member 19l (*RELT*) and beta-2-microglobulin (*B2M)* were densely connected to many other nodes (i.e., degree above 30). The detailed subnetwork signature is reported in Supplementary Table S4.

Incorporating tissue-specific mRNA expression levels of protein-coding genes in the heart, arteries, kidneys, liver and lungs (see Methods), we carried out biological pathway enrichment of the proteins in our signature for each tissue type, separately. MACE predictive proteins over-expressed in the heart were related to cell chemotaxis, cell development, ECM organization, involved in apoptotic signalling pathway, muscle structure development and homeostatic process (Figure 3B). Those over-expressed in the arteries largely overlapped with those expressed in the heart, with enrichment in cell projection organization, response to hormone, peptidyl-tyrosine phosphorylation and transmembrane receptor protein tyrosine kinase signalling pathway. Proteins specifically expressed in the liver were found to be enriched in inflammatory response and cytokine-mediated signalling pathways, as expected (Supplementary Table S5).

Using the echocardiographic variables and natriuretic peptides as endophenotype, the visualization of their first-degree neighbors in Figure 3C highlights plasma proteins worthy of further investigation, many of which are known biomarkers in various cardiovascular diseases. All network edges had positive *d-scores* (in red), suggesting that the combined co-expression scores were higher in the MACE group compared to event-free group. Among the eight echocardiographic imaging parameters in the signature, E/e' was most connected to plasma proteins, including parathyroid hormone (*PTH*), *EFEMP1*, *FSTL3*, *RSPO4*, *THBS2* and *SPON1*. The pair with the highest discriminatory power and *d-score* was between the ratio E/e' with *EFEMP1*, followed by LV mass with *FSTL3*. BNP was connected to LV mass, LA area, LVEF, LVIDSi and E/e'; ANP was only connected with LV mass. NT-proBNP was connected to key proteins such as growth differentiation factor 15 (*GDF15*), *EFEMP1*, *BMP10*, *IGFBP2* and *RSPO4*; NT-proANP was connected to the same proteins except *RSPO4* and to additional proteins including *B2M*, *CAPG*, *TFF3* and *FSTL3*.

### Predictive subnetwork signature of HF

Next, we repeated the supervised analysis for the subgroup of patients who developed HF. iOmicsPASS + identified 566 edges in the HF signature with a lower mean cross-validated error of 26.7%. This subnetwork consists of 164 proteins, 8 lipids, 12 echo imaging parameters and 5 clinical biomarkers, all of which largely overlapped with the MACE signature (Table 2). Not surprisingly, echocardiographic variables contributed more to the signature with higher *d-values* in the prediction of HF. In addition to the eight variables in the MACE signature, four additional echocardiographic variables (LA width, LVEDVi, LVIDDi and a') were included in the HF signature. hsTNI, seven proteins including interleukin-1 receptor-like 1 (*IL1RL1*), *POSTN*, *CD55* and *CD93*, two lipids (PE(O-36:4), PC(*P*-30:0)) and acylcarnitine C14:0 were also unique to this signature.

**Figure 4:**
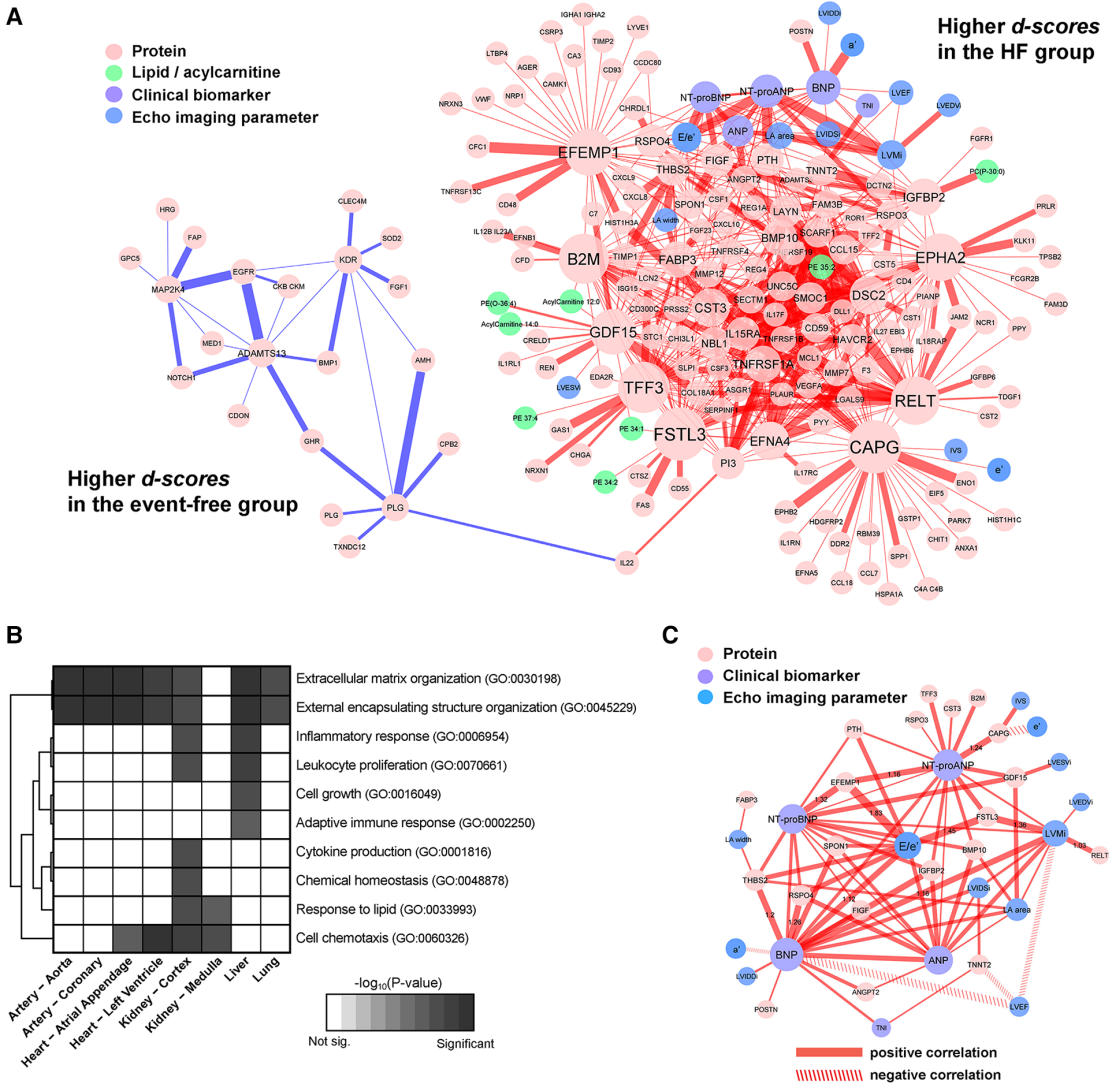
Subnetwork signature of HF. (**A**) The edges were colored by the sign of the *d-scores* for the HF group (positive in red, and negative in blue, respectively). The thickness of edges reflects the magnitude of the scores. (**B**) Enrichment of biological functions in the constituent nodes in the panel (**A**) accounting for tissue-specific expression of genes at the mRNA level. (**C**) A subset of the HF signatures consisting of first-degree neighbors of echocardiographic variables and clinical biomarkers, with line properties of the edges set according to the signs of respective partial correlations (positive in solid, and negative in dashed).

Similarly, the HF subnetwork signature showed two contrasting segments, one with higher centroids for HF (red edges) and the other with lower scores (blue edges) compared to event-free patients (Figure 4A). Most edges connected one protein to another (84.1%), 34 edges were between natriuretic peptides and proteins, 18 were between echo imaging parameters and proteins, and 12 were between lipids and proteins. Interestingly, the edges with the highest *d-values* for HF did not involve NT-proBNP. Instead, *d-scores* between *CAPG* and *FSTL3*, *TFF3*, *B2M* and *EPHA2* (*d-scores* between 1.60 to 1.89) and between *EFEMP1* and *FSTL3*, *GDF15*, *B2M*, *RSPO4*, *BMP10* and E/e' (*d-scores* between 1.60 to 1.85) were higher than that between NT-proBNP and *EFEMP1* (*d-score *= 1.32). The ratio E/e', a validated indicator of LV filling pressures, was also connected with other proteins such as *FSTL3*, *RSPO4*, *THBS1*, *PTH*, *SPON1* and the four natriuretic peptides, illustrating its joint prognostic value in predicting future HF. Full results are reported in Supplementary Table S6.

Proteins in the signature with respective mRNA over-expression in the arteries and the heart were enriched for ECM organization, external encapsulating structure organization and an additional cell chemotaxis in the heart only. Proteins over-expressed in kidney were pro-inflammatory response, lipid response, cytokine production and chemical homeostasis, whereas the proteins of hepatic origin were related to adaptive immune response and cell-growth (Figure 4B). The results of the tissue-specific enrichment analyses are reported in Supplementary Table S7.

Focusing on the subnetwork signature involving the echocardiographic variables, natriuretic peptides and hsTNI, we visualized the important proteins connected with each marker (Figure 4C). Both ANP and NT-proANP were connected to *BMP10*; both BNP and NT-proBNP were connected to *THBS2*, *RSPO4*, *IGFBP2*, *VEGFD/FIGF* and *SPON1*. Only one protein *IGFBP2* was strongly connected to all four natriuretic peptides. Among the echocardiographic measurements, other than E/e', LV mass connected with all four natriuretic peptides and two proteins (*FSTL3*, *RELT*). On the other hand, the ratio of LVEF to BNP, LV mass and *TNNT2* (i.e., negative correlation) yielded higher *d-scores* for the HF group when compared to the event-free group.

### Characterization of plasma protein markers by potential tissues of origin

**Figure 5:**
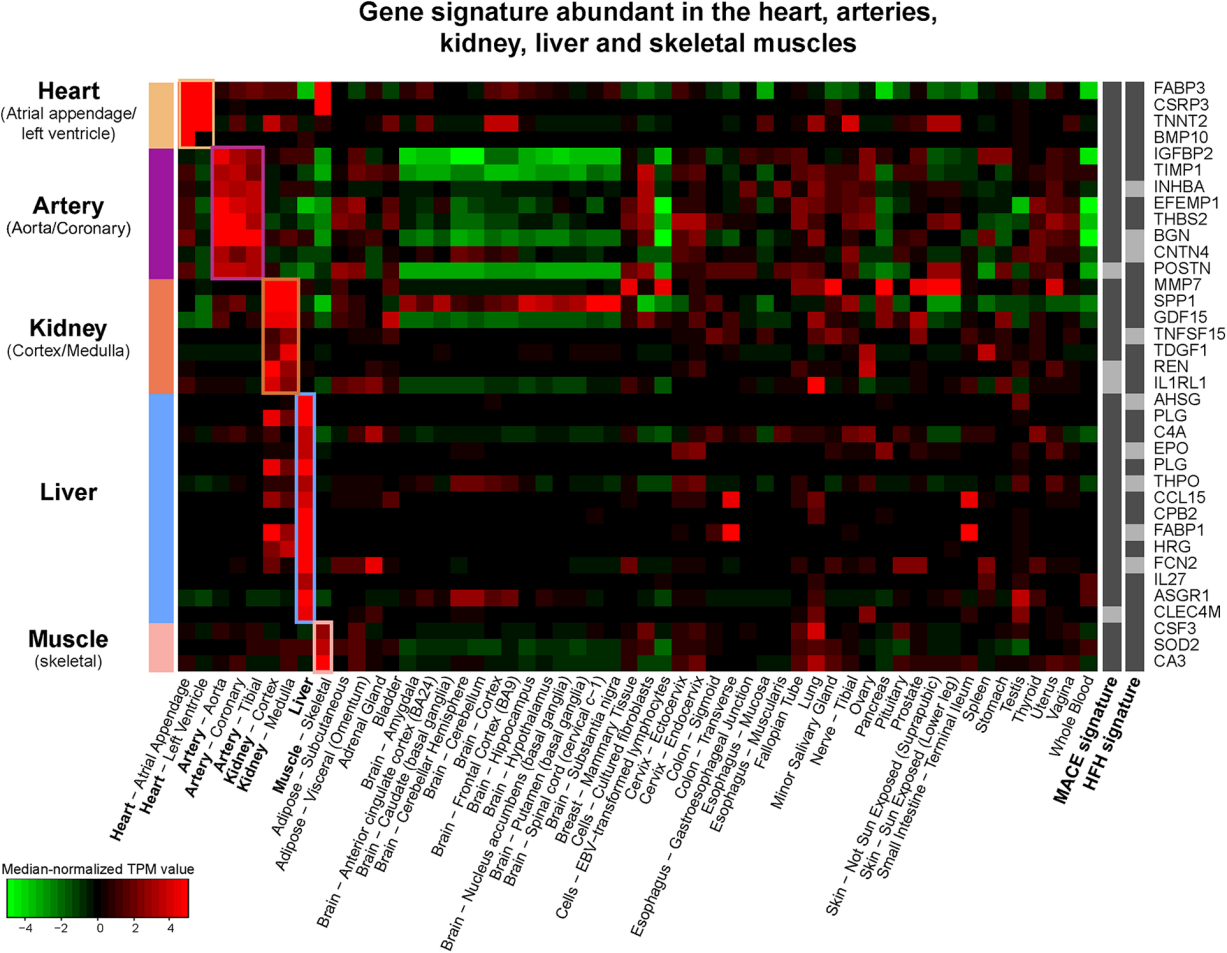
Tissue specific gene expression (median-normalized TPM values) of 36 plasma proteins in the MACE and HF signatures with specific enrichment in heart, arteries, kidneys, liver, and skeletal muscle according to the GTEx database.

To delineate the proteins directly associated with cardiac assault and tissue damage post-MI and those representing other systematic responses, we mapped the proteins to the tissue-enriched genes in the transcriptomic data provided by the Genotype-Tissue Expression (GTEx) database. Based on our definition of tissue-enriched genes (see Methods), 63.4% of the proteins in the secondary MACE signature and 66.5% of the proteins in the HF signature were enriched in at least one of the 54 tissues catalogued in the GTEx. Figure 5 shows the median normalized gene expression (in transcripts per million, TPM) of the tissue-enriched markers from the two signatures, illustrating the specificity of hub proteins to the five tissues related to the heart, arteries, kidneys, liver and skeletal muscle.

In the MACE signature, 10.6% of proteins were specifically of hepatic origin, followed by lungs (9.8%) and arteries (5.7%). In the HF subnetwork signature, the majority were enriched in lungs (13.8%), followed by liver (8.3%) and kidney (5.5%) (Table 2). In both signatures, only four proteins were enriched in the heart tissues: *BMP10*, *TNNT2*, cysteine and glycine rich protein 3 (*CSRP3*) and hFABP (*FABP3*). Among the proteins enriched in the aortic and coronary arteries, *EFEMP1*, *IGFBP2*, *THBS2*, metalloproteinase inhibitor 1 (*TIMP1)*, were shared between both signatures; biglycan (*BGN)*, contactin-4 (*CNTN4)* and inhibin beta A chain (*INHBA)* were unique to the MACE signature, while *POSTN* was unique to HF signature. The table of tissue-enriched proteins is reported in Supplementary Table S8.

### Predictive performance of subnetwork signatures in IMMACULATE cohort

Next, we assessed the predictive value of the two subnetwork signatures in 190 post-MI patients from the IMMACULATE registry, applying the same inclusion criteria. In IMMACULATE, we did not remove any patients with self-reported history of MI due to the small number of MACE cases and the information regarding how long ago the episode took place was not available. This cohort was recruited between 2011 and 2014 in Singapore (median follow-up period 4.2 years). The patients in IMMACULATE were substantially younger (mean = 53.3 ± 8 years) than those in CDCS, with a much larger proportion of current smokers (63.2%) compared to only 6.7% in CDCS and, majority of the patients were STEMI in IMMACULATE (53.5%) and NSTEMI in CDCS (70.3%). Thus, both cohorts represent post-MI patient populations from two different time periods with substantially different ethnic and genetic background and more contemporary clinical management during the follow-up in the latter. More details of the characteristics of IMMACULATE patients are provided in Table 3 and the comparison of the echocardiographic variables between event-free group and MACE group as well as HF group in IMMACULATE study is reported in Supplementary Table S9.

**Table 3:** Clinical characteristics of post-AMI patients in IMMACULATE study.

	IMMACULATE (Singapore)			
	All	Event-free	MACE	*P*-value
	(*n* = 190)	(*n* = 152)	(*n* = 38)	
**Follow-up time, median (yrs)**	4.19	4.32	2.07	
**Time to MACE, median (yrs)**		–	0.98	
**Age (yrs), mean (SD)**	53.3 (8)	53 (7.7)	54.7 (9.1)	0.258
**BMI (kg/m^2^), mean (SD)**	25.6 (4)	25.6 (3.7)	25.5 (4.7)	0.949
Ethnicity, *n* (%)				
*Chinese*	105 (55.3)	88 (57.9)	17 (44.7)	0.082
*Malay*	44 (23.2)	30 (19.7)	14 (36.8)	
*Indians*	41 (21.6)	34 (22.4)	7 (18.4)	
Smoking status, *n* (%)				
*Current Smoker*	120 (63.2)	90 (59.2)	30 (78.9)	0.077
*Ex-Smoker*	19 (10)	16 (10.5)	3 (7.9)	
*Never Smoked*	51 (26.8)	46 (30.3)	5 (13.2)	
ST-elevation status, *n* (%)				
*ST-elevated MI, STEMI*	105 (55.3)	81 (53.3)	24 (63.2)	0.362
*Non ST-elevated MI, NSTEMI*	85 (44.7)	71 (46.7)	14 (36.8)	
**Family history of CAD, *n* (%)**	37 (19.5)	33 (21.7)	4 (10.5)	0.169
**Diabetes Mellitus, *n* (%)**	32 (16.8)	25 (16.4)	7 (18.4)	0.961
**Hypertension, *n* (%)**	75 (39.5)	61 (40.1)	14 (36.8)	0.853
**Hyperlipidemia, *n* (%)**	77 (40.5)	64 (42.1)	13 (34.2)	0.483
Medication use at discharge, *n* (%)				
*Beta-blockers*	155 (81.6)	123 (80.9)	32 (84.2)	0.815
*ACE-inhibitors*	106 (55.8)	87 (57.2)	19 (50)	0.535
*Aspirin*	186 (97.9)	148 (97.4)	38 (100)	0.585
*Angiotensin receptor blockers*	17 (8.9)	14 (9.2)	3 (7.9)	1.00
*Statins*	189 (99.5)	151 (99.3)	38 (100)	1.00
Clinical biomarkers^†^, mean (SD)				
*High-sensitive Troponin I (hs-TNI), ng/l*	55.9 (235.4)	35.6 (163)	137 (407.7)	0.0173
*Atrial natriuretic peptide (ANP), pg/ml*	9.73 (0.7)	9.65 (0.7)	10.1 (0.7)	<0.001
*Brain natriuretic peptide (BNP), pg/ml*	14.1 (1.3)	13.9 (1.2)	14.7 (1.2)	<0.001
*N-terminal pro BNP, pg/ml*	701 (712.7)	591 (551.4)	1,140 (1052.4)	<0.001

^†^ Markers were measured one month from hospital discharge post MI.

In IMMACULATE, 38 patients had secondary MACE events, of which 23 were HF, representing a lower frequency of secondary MACE than CDCS, although there may be under-reporting of MACE as hospitalizations as unstable angina status was not collected. Despite these differences and in the absence of NT-proANP and ANP, the prognostic value of NT-proBNP as a single marker of MACE and HF remained exceptionally high. Supplementary Figure S2 clearly shows that NT-proBNP stratifies post-MI patients into three groups of well-separated risks (log-rank test *P*-values < 0.01) based on tertiles in both studies.

**Figure 6:**
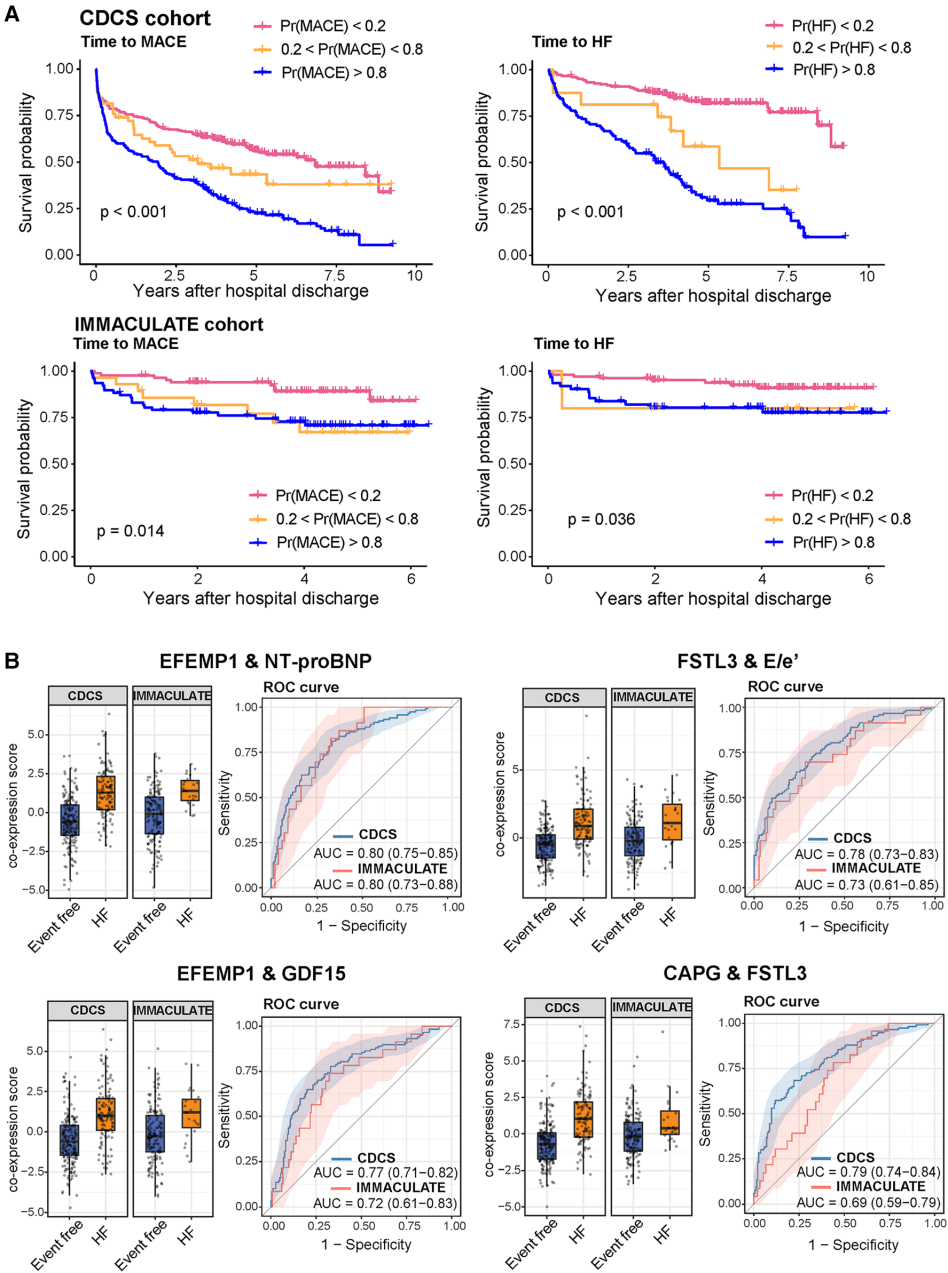
Risk stratification by the iOmicsPASS probability scores of future MACE and HF events in the cohorts. (**A**) Kaplan-Meier curves with stratification of patients by three categories based on the probability scores of MACE and HF in the training dataset (CDCS) and the validation dataset (IMMACULATE). (**B**) Boxplots of the co-expression scores of the four top scoring feature pairs predictive of HF and their respective ROC curves.

To see whether the incorporation of all other correlated molecular markers improves the predictive performance, we used the subnetwork signatures derived in the CDCS study to calculate the classification probability scores of MACE and HF for the IMMACULATE subjects. While we observed that the survival curves of high and low risk groups were well separated for MACE (*P* = 0.014) and HF (*P* = 0.036), the separation of survival curves showed at most a comparable separation of outcome groups to the results using NT-proBNP (Figure 6A). This observation was a direct testament to why natriuretic peptides have long overruled the discovery of new circulating biomarkers for incident cardiovascular events.

However, our predictive analysis approach to this data was to discover what other molecular and imaging parameters underlie the variable risk strata beyond NT-proBNP and what other biological processes are reflected in the circulation. Since iOmicsPASS + reports edge-wise co-expression values for pairs of data features in every patient, we were able to dissect the prognostic signals into network edges and examined their individual contributions to the prediction. In particular, we examined 41 pairs of data features with *d-scores* above 1.2 in absolute value (arbitrarily chosen threshold) and compared the AUC values in both CDCS and IMMACULATE. Of these, Table 4 shows that the top ten pairs of data features that had the highest AUC values above 0.73 without natriuretic peptides and up to 0.80 with NT-proBNP. All 11 proteins involved in these connections were network hub features: ECM-associated proteins *EFEMP1*, *CAPG*, *FSTL3*, and imaging parameter E/e'. Similar to the CDCS cohort, we discovered that the joint predictive powers of most of these pairs, as measured by the AUC value, were comparable to that of NT-proBNP in IMMACULATE. However, a few others such as the pair of *FSTL3* and *CAPG* were not as reproducible between the two populations (Figure 6B), which explains the lack of improvement of the subnetwork signature in AUC values over the dominant NT-proBNP as a single marker.

**Table 4:** The predictive performance and hazard ratios of the top ten pairs of data features with the highest AUC and largest magnitude of *d-scores* for HF in the CDCS and IMMACULATE cohorts.

Feature A	Feature B	DataType A	DataType B	*d-score* for Event-free	*d-score* for HF	CDCS study				IMMACULATE study			
						AUC (95% CI)	HR	*P*-value	Harrell's C-index	AUC (95% CI)	HR	*P*-value	Harrell's C-index
EFEMP1	NT-proBNP	Protein	Biomarker	−1.538	1.319	0.80 (0.75–0.85)	1.78	<0.001	0.764	0.80 (0.73–0.88)	1.92	<0.001	0.785
CAPG	FSTL3	Protein	Protein	−2.206	1.891	0.79 (0.74–0.84)	1.49	<0.001	0.734	0.69 (0.59–0.79)	1.38	0.001	0.679
FSTL3	E/e'	Protein	Imaging	−1.690	1.449	0.78 (0.73–0.83)	1.49	<0.001	0.727	0.73 (0.61–0.85)	1.62	<0.001	0.734
EFEMP1	E/e'	Protein	Imaging	−2.129	1.825	0.78 (0.72–0.83)	1.58	<0.001	0.732	0.71 (0.60–0.83)	1.52	0.001	0.718
CAPG	TFF3	Protein	Protein	−2.179	1.868	0.78 (0.72–0.83)	1.45	<0.001	0.721	0.68 (0.57–0.79)	1.34	0.002	0.659
EFEMP1	FSTL3	Protein	Protein	−2.161	1.853	0.77 (0.72–0.83)	1.52	<0.001	0.734	0.74 (0.64–0.84)	1.47	<0.001	0.725
RSPO4	BNP	Protein	Biomarker	−1.469	1.259	0.77 (0.72–0.83)	1.60	<0.001	0.723	0.77 (0.68–0.87)	1.70	<0.001	0.768
CAPG	CST3	Protein	Protein	−1.539	1.320	0.77 (0.72–0.83)	1.46	<0.001	0.721	0.59 (0.47–0.71)	1.20	0.106	0.577
EFEMP1	GDF15	Protein	Protein	−1.981	1.698	0.77 (0.71–0.82)	1.57	<0.001	0.736	0.72 (0.61–0.83)	1.45	0.001	0.695
CAPG	B2M	Protein	Protein	−1.963	1.683	0.77 (0.71–0.82)	1.44	<0.001	0.720	0.61 (0.49–0.74)	1.27	0.029	0.595

Taken together, NT-proBNP remains the most predictive biomarker of secondary MACE and HF in this study. However, a combination of two ECM proteins (*EFEMP1* and *FSTL3*) and E/e' proved to be powerful predictors of MACE and HF risk, and both protein markers can be explored as potential therapeutic targets in post-MI patients given the emerging evidence (27–30).

### Prognostic value of the network signatures by adjusting for clinical variables

Our network-based supervised method constructs a binary classifier without considering the event times and censored outcomes, and we purposefully ruled out other clinical variables in the main analysis. Nonetheless, we were able to demonstrate that the classification probability scores of MACE and incident HF using molecular data and echo imaging information showed significant differences in the Kaplan-Meier curves among the three risk groups. We next sought to determine if the prognostic value of the classification probability scores could be further improved by adjusting for clinical variables. We calculated the HRs per 10% increase in the probability scores for MACE and HF and evaluated if adding clinical variables improves the overall cNRI and IDI (Table 5).

**Table 5:** The hazard ratios (HR), C-index, continuous net reclassification index (cNRI) and the integrated discrimination index (IDI) of classification probability scores from MACE and HF network signatures, adjusting for clinical variables and medication use at hospital discharge.

	Hazard Ratio HR (95% CI)	Harrel's C-index (95% CI)	cNRI^#^	*p*-value	IDI^#^	*p*-value
Major adverse cardiovascular events (MACE)						
CDCS						
Unadjusted Model A^†^	1.09 (1.06–1.12)	0.60 (0.56–0.64)	–		–	
Adjusted Model B^‡^	1.10 (1.07–1.13)	0.50 (0.46–0.54)	0.013	0.37	0.002	0.10
Adjusted Model C^§^	1.07 (1.04–1.10)	0.49 (0.46–0.53)	0.098	0.01	0.017	<0.01
IMMACULATE						
Unadjusted Model A^†^	1.11 (1.03–1.20)	0.63 (0.54–0.71)	–		–	
Adjusted Model B^‡^	1.12 (1.03–1.21)	0.62 (0.53–0.70)	0.021	0.43	0.004	0.27
Adjusted Model C^§§^	1.13 (1.04–1.22)	0.66 (0.57–0.74)	0.234	0.08	0.017	0.03
Incident heart failure (HF)						
CDCS						
Unadjusted Model A^†^	1.19 (1.14–1.24)	0.74 (0.69–0.79)	–		–	
Adjusted Model B^‡^	1.15 (1.10–1.21)	0.43 (0.37–0.48)	0.131	0.13	0.028	0.05
Adjusted Model C^§^	1.12 (1.06–1.18)	0.55 (0.49–0.60)	0.22	0.02	0.043	<0.01
IMMACULATE						
Unadjusted Model A^†^	1.12 (1.03–1.22)	0.70 (0.62–0.77)	–		–	
Adjusted Model B^‡^	1.12 (1.02–1.22)	0.69 (0.59–0.78)	0.073	0.50	0.006	0.23
Adjusted Model C^§§^	1.11 (1.01–1.22)	0.73 (0.63–0.81)	0.394	0.06	0.115	<0.01

^†^ Model is not adjusted for any other factors.

^‡^ Model is adjusted for age, gender and BMI. For IMMACULATE, gender is omitted since all patients are male.

^§^ Model is further adjusted for ST elevation status at admission, medical history of hypertension and use of beta-blockers, ACE inhibitor, aspirin, clopidogrel, calcium channel antagonist, long-acting nitrate, diuretics, statin and warfarin at hospital discharge.

^§§^ Model is further adjusted for ST elevation status at admission, medical history of hypertension and use of beta-blockers, ACE inhibitor and antagonist receptor blocker at hospital discharge.

^#^ cNRI and IDI are calculated based on 2-year survival and the unadjusted model A is used as the base model.

In CDCS, the HR per 10% increase in the probability score is associated with a HR of 1.09 (95% CI: 1.06–1.12) and 1.19 (95% CI: 1.14–1.24) times increase in risk of MACE and HF, respectively. After adjusting for age, gender and BMI, the HR becomes 1.10 (95% CI: 1.07–1.13) and 1.15 (95% CI: 1.10–1.21) for MACE and HF, respectively. The adjusted models did not improve the model's performance with low values of cNRI and IDI (*p*-value ≥ 0.05) for both outcomes. However, after further adjusting for ST-elevation status, hypertension and medication use at discharge, the adjusted HRs became 1.07 (95% CI: 1.04–1.10) for MACE and 1.12 (95% CI: 1.06–1.18) for HF, with significant improvement in reclassification for both outcomes (*p*-value < 0.05). Similarly for IMMACULATE, the fully adjusted HRs were 1.13 (95% CI: 1.04–1.22) for MACE and 1.11 (95% CI: 1.01–1.22) for HF and only the fully adjusted model showed significant improvement from the unadjusted model with significance for IDI (*p*-value < 0.05) and marginal significance for cNRI (*p*-value < 0.10). The results are summarized in Table 5.

## Discussion

We used interpretable machine learning to connect the global landscape of plasma proteins and lipids with echocardiographic imaging variables and established circulating biomarkers in two independent patient cohorts hospitalized for AMI with heterogeneous and contrasting ethnic and genetic background. Plasma proteins carried stronger prognostic signals than lipids in both cohorts, and communities of plasma proteins were associated with increased risks of long-term secondary MACE and HF. However, we strongly suspect that the lower prognostic potential of lipids is largely affected by the nearly universal prescription of statins at hospital discharge. Notably, we identified new prognostic plasma proteins and echocardiographic variables with equivalent classification performance to existing biomarkers, with different biological and mechanical interpretation. The diastolic parameter E/e' outperformed LVEF as a prognostic variable, while two network hub proteins *EFEMP1* and *FSTL3* maintained their prognostic significance similar to NT-proBNP and other established circulating biomarkers. As blood is frequently the only available reporter tissue in CVD biomarker discovery, we also annotated individual proteins in terms of their potential tissue(s) of origin. Interestingly, the number of prognostic proteins of liver, lung and kidney origin outperformed those specific to the heart and arteries.

The network hub proteins with reproducible signals in the predictive subnetworks were *EFEMP1* and *FSTL3*. *EFEMP1*, also known as fibulin-3, is an ECM glycoprotein implicated in vascular endothelium remodeling (31). It plays an important role in reducing vascular calcification and inhibiting metalloproteinases in oxidative stress (32–34). *FSTL3*, an extracellular regulator of TGF-*β* family cytokines such as activin A, is involved in various biological functions including cell proliferation and inflammation, and altered transcriptomic regulation of the gene with another follistatin family member *FSTL1* in myocardium has been associated with HF severity (30). *FSTL3* has also been characterized as a stress-induced regulator of cardiac hypertrophy through Smad signalling pathway modulation in a mouse model (35), opening an opportunity for investigation of the follistatin family of proteins as a therapeutic target for the protection against HF.

Despite the lower prognostic power of lipids, both subnetwork signatures included four lipid transport proteins, namely *APOL1*, *APOE*, *APOA1* and *FABP3*. *FABP3* encodes hFABP, a marker of myocardial injury. hFABP was connected to a multitude of acylcarnitine species in both subnetwork signatures. It has previously been implicated in HF, where reduced fatty acid utilization in the heart leads to the progression of chronic HF, left ventricular hypertrophy and remodelling (36, 37). Intriguingly, here we found that circulating levels of hFABP and acylcarnitines (C12:0, C14:0, C14:1, C14:2, C16:1) were jointly increased in MACE and HF patients, suggesting that the circulating levels may have a dual role in both ischemic and HF events.

One of the most challenging issues in analyzing plasma molecular profile, especially among patients with predisposed conditions, is the use of medications and combination of drugs prescribed during the treatment of the primary episode, which could potentially confound the outcomes of interest and/or affect the protein or lipid levels in the blood. In both our studies, all patients were prescribed at least one type of medications at hospital discharge for the treatment of the primary AMI and most patients were on beta-blockers, aspirins and statins in both CDCS and IMMACULATE study. Thus, it is difficult to truly evaluate if the medication use to blood collection at baseline could potentially mediate the outcomes or affect the molecular profile. To this end, we investigated the medication use by the patients during hospital admission and discharge in both cohorts.

We first identified the types of medications directly associated with secondary MACE and HF. In CDCS, the use of beta-blockers, antiplatelet therapy (clopidogrel), calcium channel antagonists, long-acting nitrates, diuretics, statins and warfarin at hospital discharge were associated with both MACE and HF. Similarly, the same medications, except clopidogrel, and two additional medications (ACE inhibitors and aspirin use) at admission were associated with both outcomes. In IMMACULATE, medication information was collected for fewer drugs, but none were associated with MACE or HF. This is likely due to the small number of secondary outcomes and the resulting lack of statistical power. Then, we fitted separate Cox PH models on individual markers to obtain the unadjusted HR and HR adjusted for the medication use at discharge. Supplementary Figure S3 shows the scatter plot of the unadjusted and adjusted HR and data features that were part of the network signatures for MACE and HF are colored as blue (protein), green (lipid), salmon (clinical biomarker) and purple (echo imaging). The plots show that the HRs before and after adjusting for medication use remain unchanged and this observation suggests that the associations we reported are unlikely to be altered by medication use. The full statistical summary can be found in Supplementary Table S10.

Overall, our study had several limitations. First, the IMMACULATE cohort had a smaller size and fewer MACE and HF events compared to the CDCS cohort, leading to borderline statistical significance of several associations in the IMMACULATE cohort. Second, the acute phase of AMI and various interventions including pharmacotherapies and revascularization may have confounded the association between data features and events; we deliberately performed the proteomic and lipidomic analysis using blood samples collected at the 30-day post-MI timepoint instead of close to the time index AMI event in order to reflect a more “steady-state” after initial treatment and stabilization, yet we acknowledge that this sampling scheme may have introduced bias from heterogeneous treatment courses of the primary AMI events across the patients. Third, the number of proteomic targets measured was larger than that of the lipid targets by almost four-fold; this might partially explain the greater prognostic signals from proteins than lipids in our study, in addition to the effects from the medications with immediate impact on circulating lipid levels. Fourth, higher resolution imaging tools including cardiac magnetic resonance could have contributed a much richer set of imaging features to the networks. Last but not least, the proteomic assay platform could have suffered from the potential cross-reactivity of the SOMAmer reagents (e.g., across multiple member proteins of the same family such as follistatin and fibulin) as cautioned by an increasing number of publications (38–40).

These shortcomings notwithstanding, our work is to the best of our knowledge the first network-based multivariate analysis of large-scale cohorts of post-MI patients to explore the interplay between gold-standard natriuretic peptides with echocardiographic imaging variables and circulating plasma proteins, lipids and acylcarnitine. With potential caveats in the choice of sampling points and the proteomic assay in mind, our analysis highlighted that there exist a plethora of under-recognized data features that not only jointly modulate the risk of post-MI HF but also open the potential for therapeutic intervention through modulation of ECM. In this study we highlighted two ECM proteins *FSTL3* and *EFEMP1*, both elevated in response to the primary AMI event. According to our inference of tissue gene expression data, it is highly likely that they are secreted from the myocardium and the microvasculature and the aorta and they participate in key cell fate decisions and damaged myocardial tissue repair during the response. Overall, with the increasing throughput of proteomic assays and the power of interpretable machine learning techniques, we are confident that more promising cues for improved risk stratification and therapeutic intervention can be discovered to mitigate HF after AMI.

## Data Availability

The original contributions presented in the study are publicly available. This data can be found here: iOmicsPASS+ on open-source R package in the GitHub repository (https://github.com/cssblab/iOmicsPASSplus). The proteomic and lipidomic data in support of the findings of this study are available at https://github.com/Hiromikwl/Data_iOP. Patient-level clinical records, including echocardiographic imaging variables and clinical biomarkers can only be shared on reasonable request due to data privacy concerns.
